# Better Movers and Thinkers (BMT): An Exploratory Study of an Innovative Approach to Physical Education

**DOI:** 10.5964/ejop.v11i4.950

**Published:** 2015-11-27

**Authors:** Andrew Dalziell, James Boyle, Nanette Mutrie

**Affiliations:** a Moray House School of Education, Institute for Sport, Physical Education and Health Sciences, University of Edinburgh, Edinburgh, United Kingdom; b School of Psychological Sciences and Health, University of Strathclyde, Glasgow, United Kingdom; Academy of Special Education, Warsaw, Poland

**Keywords:** physical education, academic achievement, executive function, activity

## Abstract

Recent research has confirmed a positive relationship between levels of physical activity and academic achievement. Some of these studies have been informed by neurological models of Executive Functioning (EF). There is a general consensus within the literature that the three core EF skills are; working memory, inhibitory control and cognitive flexibility. The development of these core EF skills has been linked with learning and academic achievement and is an essential component in the delivery of PE using a new and innovative approach called ‘Better Movers and Thinkers (BMT).’ A mixed methods design was used to investigate the effectiveness and feasibility of a 16-week intervention programme using BMT where 46 children were tested on two separate occasions for coordination and balance control, academic skills, working memory and non-verbal reasoning skills. One school acted as the control condition (21 students, aged 9 – 10 years) and another school acted as the intervention condition (25 students, aged 9 – 10 years). Quantitative data revealed an effect between pre and post-test conditions in the areas of phonological skills (p = .042), segmentation skills (p = .014) and working memory (p = .040) in favour of the intervention condition. Further analysis identified a gender-interaction with male students in the intervention condition making significant gains in phonological skills (p = .005) segmentation skills (p = .014) and spelling (p = .007) compared to boys in the control condition. Analysis of qualitative data from a sample of students from the intervention condition and their class teacher indicated good acceptability of BMT as an alternative approach to PE.

## Introduction

Recent research has reignited interest into the physical and cognitive benefits of physical activity with some studies specifically highlighting beneficial aspects of activity on Executive Functioning (Blair & Razza, 2007; Coe, Pivarnik, Womack, Reeves, & Malina, 2006; Diamond & Lee, 2011; Trudeau & Shephard, 2010). Executive Functioning (EF) is an umbrella term that describes the complex cognitive processes required to perform novel or difficult goal-directed tasks, including the ability to delay or inhibit responses, develop a plan of action sequences, and working memory. Recent research has suggested plausible links between physical activity and the enhancement of EF including; physiological influences such as greater cerebral blood flow; increased secretion of neurotrophins as a result of increasing levels of physical activity; psychological influences such as an increase in self-esteem and a desire to learn and be part of the school (Ahn & Fedewa, 2011; Strong et al., 2005; Trudeau & Shephard, 2010). Other studies have identified that no decrease in academic performance has been observed despite a curtailing of time spent teaching academic subjects in favour of more time participating in PE (Ahamed et al., 2007; Trudeau & Shephard, 2010). Aside from the cognitive gains that can be attributed to increasing time allocated to PE (and consequently being physically active), there is a well-established link between increasing levels of physical activity and the general health of children (Ahn & Fedewa, 2011; Kristensen et al., 2010).

Studies have investigated the impact of gentle, vigorous and chronic bouts of exercise on academic performance (Coe, Pivarnik, Womack, Reeves, & Malina, 2006; Davis & Cooper, 2011). One study carried out in the United States reported an immediate increase in concentration levels in grades 2 to 4 following 15 minutes of stretching and walking (Caterino & Polak, 1999). Whilst this demonstrates a positive impact between the engagement in physical activity and concentration levels, the activities carried out in this study are not normally part of a PE lesson and lasted for a much shorter duration. Other larger-scale studies (Hamre & Pianta, 2001; Hughes & Graham, 2002; Welsh & Pennington, 1988) have shown that being physically active is known to increase an individual’s immediate level of arousal through an increase in neural activity in the reticular formation of the brain, although the long-term impact of this increase is less established (Biddle & Asare, 2011). Similarly, endurance exercise (a sustained period of running and swimming, for example) leads to a substantial increase in systemic blood pressure where the overall perfusion of the brain typically increases by 14 – 25% (Goswami, 2008; Hamre & Pianta, 2001). It would appear that the intensity, duration and frequency of physical activity may impact differently on an individual’s potential gains with respect to their academic achievement. There is little information on the different impact that comes from different modes of activity and further research is required in this area.

Despite discussions around the diverse nature of physical activity and how this may impact differently on academic achievement, the literature reveals two related findings. Firstly, that increasing the time spent on PE and thus reducing the time spent on teaching academic subjects does not reduce academic achievement, and secondly, that when students are more physically active, this can often be associated with improvements in their academic achievement suggesting that there may be a link between physical activity and learning. These findings seem to correlate and do not appear to be influenced by variability in study design or by the different measurement techniques that are cited between studies, perhaps adding weight to the justification for increasing time allocated to PE provision in our schools.

Research further reveals divergent findings regarding the relationship between gender and academic achievement with some evidence favouring boys and some evidence girls (Hyde, 2005; Machin & Pekkarinen, 2008). Other authors (Matthews, Ponitz, & Morrison, 2009; Weaver-Hightower, 2003) note that historically boys were largely advantaged in the school classroom and most academic settings. Other studies, however, reveal that girls tend to build better relationships with their teachers, attain higher results, achieve higher levels of education and generally progress scholastically better when compared with boys (Duckworth & Seligman, 2006; Ready, LoGerfo, Burkam, & Lee, 2005; Silverman, 2003). However, within these studies there is a clear differential effect between gender depending on the subject being assessed and the nature of that assessment. The literature indicates a significant advantage for girls in language based tasks (Duckworth & Seligman, 2006) and an advantage for boys in standardised tests that may be based on their motivation within a competitive environment being greater than girls (Gneezy, Niederle, & Rustichini, 2003). One of the plausible causes underpinning these gender differences is that strong behavioural regulation developed in the earlier years in the school sets precedence for successful academic achievement through increased school engagement and motivation (Fredericks, Blumenfeld, & Paris, 2004; Zimmerman & Schunk, 2011) and studies have shown that girls are able to regulate their behaviours earlier than boys (Fredericks, Blumenfeld, & Paris, 2004; Zimmerman & Schunk, 2011). To varying degrees, self-regulation tasks tap executive functions such as attention and inhibitory control which, according to some researchers, support self-directed classroom behaviours (Blair, 2002; Brook & Boaz, 2005; Howse, Lange, Farran, & Boyles, 2003; Ponitz et al., 2008; Saracho & Spodek, 2007). Deficiencies in self-regulation present at a younger age may undermine academic achievement and predict outcomes (Green & Francis, 1988; Vitaro, Brendgen, Larose, & Trembaly, 2005) with one study suggesting that there is a particular link between inhibitory control (for example, delayed gratification, impulse control) and phonological awareness (for example, blending and segmenting of sound components and syllables within written and/or oral tasks) (Blair & Razza, 2007). This particular finding is of considerable relevance as research has shown that blending and segmentation of sounds and phonics has the greatest transfer to emergent reading and spelling (Ehri et al., 2001). Phonological awareness is known to develop earlier in girls with concomitant findings demonstrating that girls are better readers than boys (Machin & Pekkarinen, 2008). It would appear from other studies that this phenomenon may be universal and the result from large-scale international comparisons of reading literacy among 10-year olds and 15-year olds also showed that girls read better than boys in a wide variety of school systems and cultural settings (Chiu & McBride-Chang, 2006; Machin & Pekkarinen, 2008).

In summary, if time spent in PE enhances EF through participation and engagement in physical activities, then this in turn may lead to better levels of inhibitory control and attention which have been directly linked to phonological awareness. The levels of enhancement and progress may differ between boys and girls.

**Figure 1:**
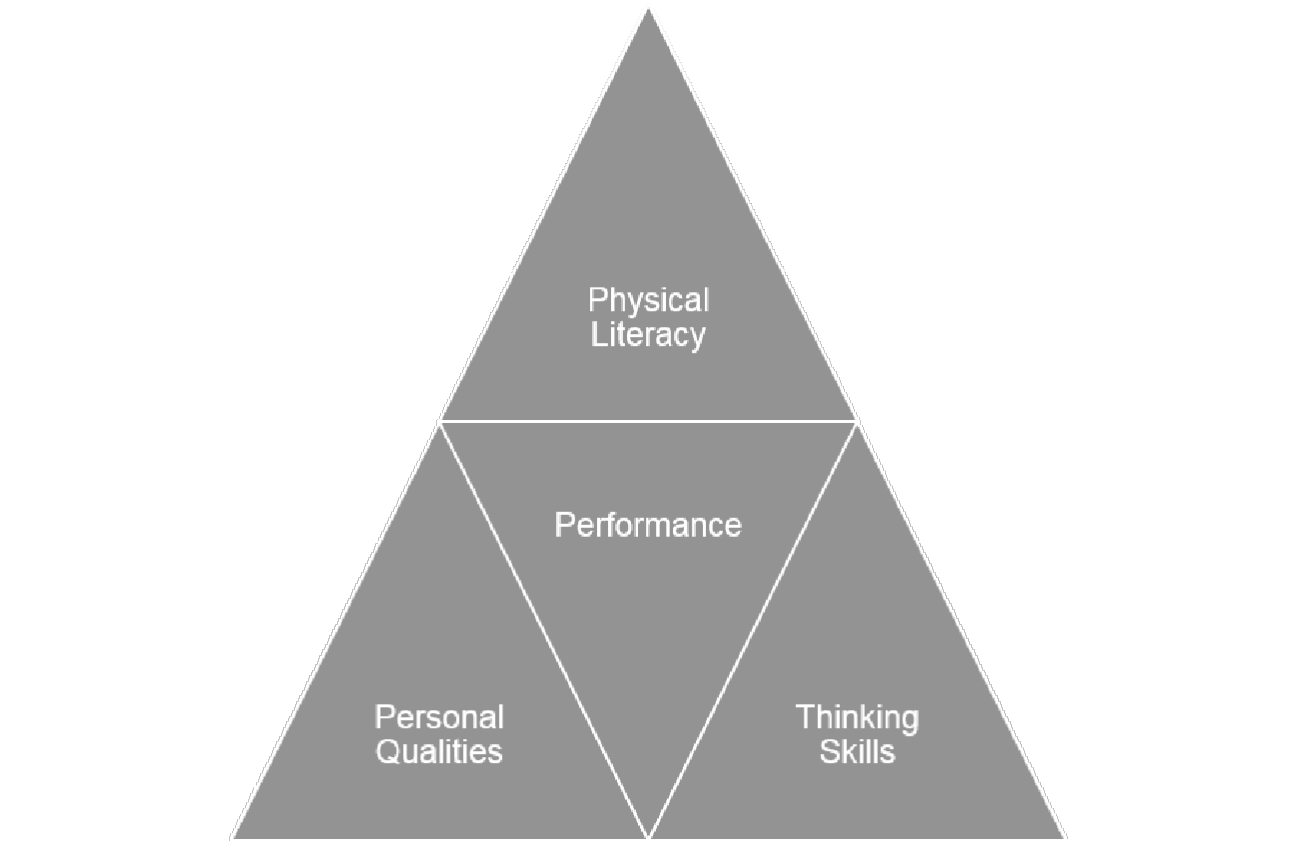
The Learning Framework.

Historically, a common approach to PE for primary age children typically involves a skills-based session which is often teacher-led (Bailey et al., 2009). An alternative approach has been developed which directly involves a shift in pedagogical practice where sports and other activities are used as a vehicle to develop three key constituent parts: thinking skills (decision making, problem solving, adaptability, working memory), human capacities (determination, perseverance, self-confidence) and physical literacy with no instruction provided with regards to the development of technical skills (see Fig. 1). This approach consciously directs a specific focus towards the inclusion of EF skills and sets out to identify if this different approach to teaching primary PE, can lead to improvements in academic achievement.

**Figure 2:**
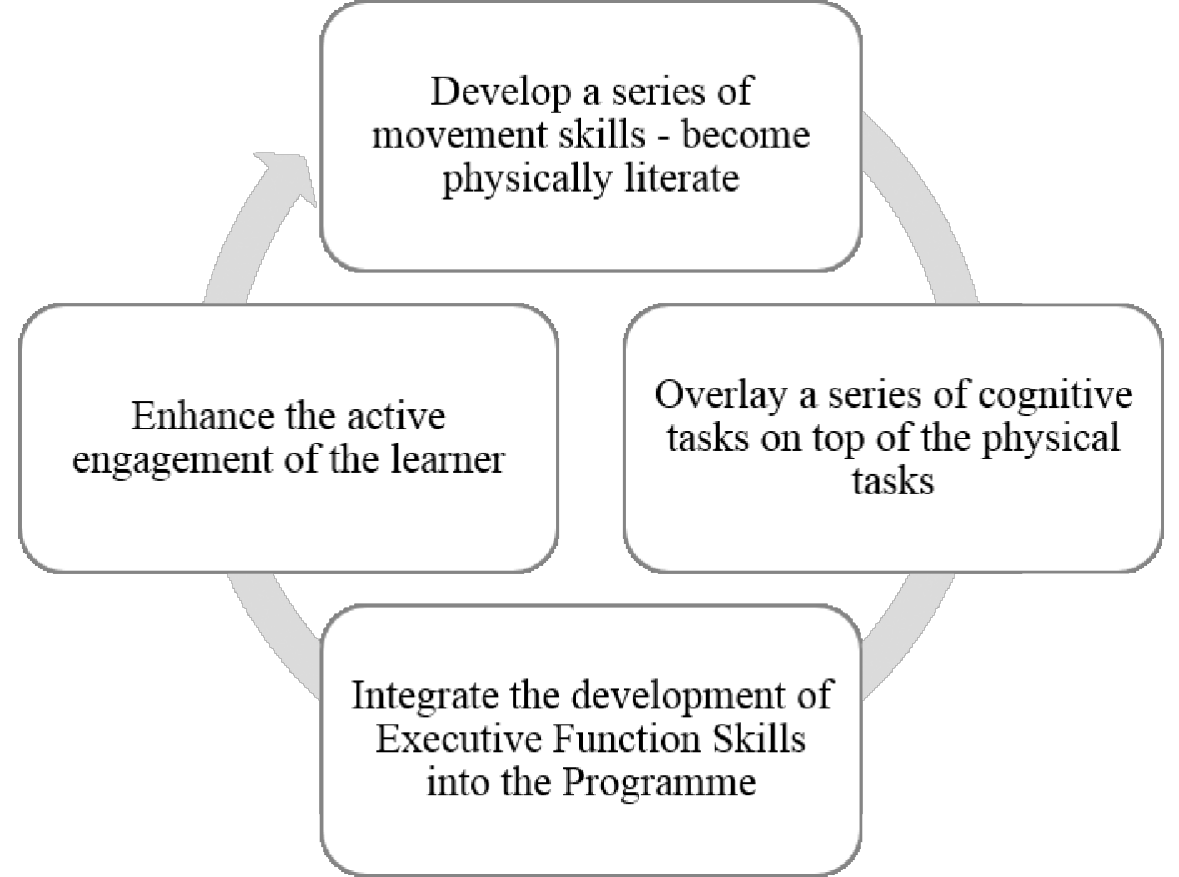
The BMT Process.

‘Better Movers and Thinkers (BMT)’ is designed to develop the ability to move and think in an integrated way within PE. Exponents of BMT contend that if children have better quality and control over their balance and movements, this can then become more automatic potentially resulting in reduced levels of conscious thought having to govern movement and balance. To coincide with this development, EF have been specifically developed through the BMT approach, assisting the development of cognitive processes, which in turn will help them, succeed across the curriculum (Diamond, Barnett, Thomas, & Munro, 2007). There have been many studies identifying the impact of good EF skills in children (Booth et al., 2013; Diamond & Lee, 2011; Koziol & Lutz, 2013; Monette, Bigras, & Guay, 2011) but there are no studies that have used PE as the context for learning. The present study therefore acts as an exploratory study into the possible links between PE and EF skill development leading to educational gains. Figure 1 represents the BMT learning framework that encompasses the three constituent parts that come together to make performance. Physical literacy focuses on the development of key physical attributes that enhance physical performance such as balance, postural control, gross motor coordination, rhythm and timing. Personal qualities relate to aspects of human characteristics such as determination, courage, motivation and perseverance which are considers by the proponents of BMT to be essential to remain engaged with the process of learning. Thinking skills refers to the development of cognitive processes such as the development, enhancement and refinement of EF skills. Figure 2 represents the BMT process that should occur in each individual session where there is the identification of the series of movement skills to develop physical literacy, the inclusion of a series of differentiated cognitive tasks to develop thinking skills, a specific focus on integrating and developing EF skills and encouraging the active engagement of the learner through the development of personal qualities.

The purpose of this study was to evaluate if BMT positively influences academic skills, and to identify what the perceptions of students and staff are of this approach within primary education. The perceptions of these stakeholders will help to inform an understanding of the acceptability, feasibility and impact of BMT in the delivery of PE within the primary school.

## Methods

### Design

A mixed methods design (Denscombe, 2008) utilising both quantitative and qualitative data was adopted for this study to provide information about the efficacy, acceptability and feasibility of BMT as an approach to delivering PE in primary schools.

### Participants

Participants in this study were aged 9 – 10 years and were attending two mainstream state schools in a Primary 6 class in Scotland, UK. Following ethical approval by the University of Strathclyde and the local authority, two schools were recruited with one hosting the intervention and the other one serving as a control. The schools were identified as being similar in terms of school roll, school facilities and were within close proximity (i.e. less than one mile) from one another. Once the schools were identified the selection of which schools acted as the control and which acted as the intervention was decided by the Quality Improvement Officer representing the local authority. Parents provided informed consent and students provided informed assent for participation and had the opportunity to opt out of the study. In the control condition, 21 students (9 boys and 12 girls) participated and in the intervention condition, 25 students (13 boys and 12 girls) participated.

### Materials

#### MABC2

Three balance subtests (One Board Balance, Walking Heel-to-Toe Forwards and Hopping on Mats) were taken from the *Movement Assessment Battery for Children-2* (MABC2) (Henderson, Sugden, & Barnett, 2007) to evaluate the movement and balance competency of each child. These 3 subtests were taken from the test due to the pragmatic limitations of running a study within school and limited access and time not being available to utilise the full scale test. Studies reveal (Croce, Horvat, & McCarthy, 2001) that the MABC2 has test-retest reliability in the range .53 - .95. The balance composite score has a high reliability (α = .90) when measuring internal consistency using Cronbach’s alphas.

#### LASS 8–11

The *Lucid Assessment System for Schools* (LASS 8–11) (Singleton, 2007) consists of 8 subtests each of which assesses a different measurable component within the three categories; EF, academic skills and non-verbal intelligence (see Table 1). The complete LASS assessment programme was undertaken by each child in both schools. The children in each school went through the subtests in the prescriptive order as outlined in the procedures of the LASS 8–11 assessor’s manual (Singleton, 2007). Z-scores were transferred into T-scores by the main researcher and used as the outcome measure to allow for a comparison to be made between conditions. The Working Memory composite score consisted of 2 items (α = .61), the Reading composite consisted of 2 items (α = .28) and the Phonological composite consisted of 2 items (α = .44). This demonstrates a moderate reliability for Working Memory, and low reliability for Phonological composite and Reading composite.

**Table 1:** Subtest Name, Category, Type and Description for LASS 8–11.

Subtest Name	Test Area
Cave	Visual spatial memory
Mobile Phone	Auditory sequential memory (digit span)
Non-Words	Phonological Awareness
Segments	Phonological decoding and encoding
Single-Word Reading	Sight word reading
Sentence Reading	Cloze reading
Spelling	Spelling
Reasoning	Non-verbal reasoning intelligence

Studies reveal that the LASS 8–11 has test-retest reliability in the range .51 - .93. This range suggests that the reliability of the assessment tools rates from fair to excellent (Cicchetti, 1994; Cicchetti & Sparrow, 1981; Fleiss, 1981) and that is suitable for this exploratory study.

### Procedures

Both schools agreed to provide two 60-minute sessions of PE each week, for 16 weeks commencing on 17^th^ January and concluding on 24^th^ May 2012. Most of the sessions took place on Monday and Thursday mornings in the control condition and Tuesday and Thursday mornings in the intervention condition. The control condition received their standard PE provision where the class teacher was supported in the delivery of PE by a PE specialist, with strategic input from National Governing Body (NGB) sports coaches. The students covered a range of activities such as team sports and gymnastics. The PE specialist, sports coaches and class teacher were unaware of the nature and content of BMT sessions throughout this study. The class teacher was aware of the study design and method.

The intervention condition received the BMT provision from two PE specialists who have contributed to the development and design of BMT from its conception. Each of these PE specialists took one session per week, and both liaised with one another each week in order to discuss how the students were performing and to establish the focus of subsequent sessions.

#### Quantitative Data

Pre and post-testing was conducted on a one-to-one basis by the main researcher and the students in both schools. Physical testing using the MABC2 was carried out with each pupil, in a quiet room within the school before completing the LASS 8–11 with each pupil on a separate day in the same quiet room. All testing was completed in December prior to the intervention commencing in January.

#### Qualitative Data

The focus group (inclusive of 8 randomly selected pupils, 4 boys and 4 girls) and classroom teacher interview were carried out by a research team assistant who was not involved in the quantitative testing protocols or in the delivery of any of the PE/BMT lessons within the control or intervention condition. The research assistant was an experienced interviewer and facilitator of focus groups.

The following three areas were covered in the pupil focus group:

1. The enjoyment levels of the students during the PE lessons
2. Pupil perceptions on what they learned in the PE lessons
3. Transferable skills from PE lessons to classroom-based learning

These three areas were selected as they would inform the study about the experience the pupils had of BMT, and would provide an insight into the links between BMT and aspects of learning as perceived by the students. This would help to further inform the efficacy and feasibility of adopting BMT as an alternative approach to PE.

The class teacher participated in an interview at the end of the study. The topics covered included:

1. Differences in classroom behaviour prior to and after the BMT/PE sessions (i.e. change in attention and concentration)
2. Changes in school attendance on the days that BMT/PE sessions were being delivered?
3. Cross-curricular links between BMT/PE and classroom learning?

### Data Analysis

#### Quantitative Data

Baseline data collection was completed prior to the start of the intervention phase and post-testing was completed within 2 weeks of the intervention phase ending. Statistical analysis was undertaken by analysis of covariance (ANCOVA) using SPSS version 19 with baseline scores as covariate.

MABC2
  Raw data from the three subtests were transferred into standard scores using a conversion table. Analyses here are based on the composite standard scores following the outlined procedures of the MABC2 assessment (Henderson, Sugden, & Barnett, 2007).
LASS 8–11
  This is a computer-based assessment tool and automatically records and presents the performance of each of the 8 subtest into raw score, centile, z-score, z-score discrepancy and age equivalent data. For the purposes of comparing results from baseline to those at the end of the 16 week intervention, the z-scores were transformed into *t*-scores for each subtest using the formula: *T = (Z score x 10) + 50*
  The working memory composite score was calculated by adding the *T*-score from the visual spatial memory subtest (i.e. Cave) to the *T-*score from the auditory sequential memory subtest (i.e. Mobile Phone) to produce an average score. Similarly, a composite score for the phonological skills was calculated by adding the *T*-score from the phonological awareness subtest (i.e. Non-Words) to the phonological decoding and encoding subtest (i.e. Segments). A composite score for reading was calculated by adding the *T*-score from the sight word reading subtest (i.e. Single Word Reading) to the cloze reading subtest (i.e. Sentence Reading) producing an average score. Composite scores were calculated using the following expression: composite = *T-score + T-score/2*

#### Qualitative Data

The first author transcribed the focus groups and class teacher interviews verbatim before analysing the results. Themes were informed by the research questions and subthemes emerged from the data. Illustrative quotes from the respondents were used to illuminate the categories. To ensure participant anonymity, pseudo names were used throughout the transcription and in the reporting of results.

## Results

21 students (9 boys, 12 girls) participated in the control condition and 25 students (13 boys, 12 girls) in the intervention condition. All of the participants presented full data for both pre- and post-test conditions.

### Quantitative Data

#### MABC2

The results from the three subtests administered revealed a ceiling effect. The highest score achievable was 19, and all students met the criteria for this score in accordance with the procedures as laid out in the assessment manual. Findings from these subtests will not be further reported.

#### LASS 8–11

Significant main effects for the intervention condition were revealed by the ANCOVA for the Working Memory Composite score (*F*(1, 41) = 4.52, *p* = .040), the Phonological Composite score (*F*(1, 41) = 4.43, *p* = .042), and the ‘Segments’ subtest (*F*(1, 41) = 6.63, *p* = .014). No significant main effects were identified for Reading Composite (*F*(1, 41) = 1.74, *p* = .195) or Reasoning (*F*(1, 41) = 0.92, *p* = .343) subtests or for Spelling, although the latter approached significance (*F*(1, 41) = 3.00, *p* = .091).

Table 2 shows the means and standard deviations for the computer-based assessment system LASS 8–11 for the control and intervention condition participants.

**Table 2:** Means and Standard Deviation (SDs) for Working Memory Composite, Phonological Skills, Reading Composite, Spelling and Reasoning for Intervention and Control Conditions Pre- and Post-Test.

Outcome Measures	Mean scores (*SD*) at Pre-Test		Mean scores (*SD*) at Post-Test	
	Control	Intervention	Control	Intervention
Working Memory	54.45 (8.22)	55.92 (5.53)	55.54 (9.41)	56.13 (5.88)
Phonological Skills	49.18 (7.56)	48.19 (7.17)	49.34 (7.07)	50.72 (6.06)
Reading Composite	58.53 (7.48)	54.04 (10.37)	51.18 (6.58)	50.64 (6.35)
Spelling	52.51 (6.97)	50.79 (7.25)	51.32 (6.50)	51.82 (6.27)
Reasoning	46.63 (7.62)	43.64 (5.67)	46.75 (7.93)	47.00 (6.15)

There were significant group by gender interaction in the case of the Phonological Composite Scores (*F*(1, 19) = 9.85, *p* = .005), the ‘Segments’ subtest (*F*(1, 19) = 10.48, *p* = .004), and ‘Spelling’ (*F*(1, 19) = 10.97, *p* = .007) with boys in the intervention condition achieving significantly higher scores than boys from the control condition in all three measures. Table 3 shows the means, standard deviations and *p*-values for the computer-based assessment system LASS 8–11 for both conditions by gender.

**Table 3:** Boys and Girls Means, Standard Deviation (SDs) at Pre- and Post-Testing for Control and Intervention Conditions.

Outcome Measures	Mean Scores (*SD*) at Pre-Test		Mean Scores (*SD*) at Post-Test		
	Control	Intervention	Control	Intervention	*p*
Working Memory					
Boys	56.20 (8.56)	57.16 (3.92)	53.57 (10.95)	57.92 (5.46)	.207
Girls	53.14 (8.06)	54.59 (6.80)	57.02 (8.26)	54.19 (5.91)	.129
Phonological Composite					
Boys	48.14 (7.62)	50.85 (5.53)	45.95 (6.07)	52.50 (3.42)	.005
Girls	49.96 (7.75)	45.31 (7.84)	51.88 (6.91)	48.80 (7.73)	.705
Non-Words					
Boys	50.02 (9.05)	49.30 (8.27)	47.60 (5.58)	50.27 (3.86)	.201
Girls	48.69 (9.64)	44.74 (7.53)	52.79 (8.26)	49.94 (9.39)	.597
Segments					
Boys	46.25 (7.70)	52.40 (8.58)	44.31 (8.08)	54.73 (5.56)	.004
Girls	51.23 (10.85)	45.87 (10.07)	50.97 (7.71)	47.66 (8.54)	.547
Read Composite					
Boys	58.76 (5.40)	52.95 (10.22)	48.82 (6.35)	51.30 (5.78)	.351
Girls	58.36 (8.97)	55.22 (10.85)	52.94 (6.44)	49.93 (7.11)	.456
Spelling					
Boys	50.69 (5.63)	52.06 (5.28)	48.56 (4.69)	53.36 (2.84)	.007
Girls	53.87 (7.78)	49.41 (8.97)	53.40 (7.06)	50.16 (8.43)	.813
Reasoning					
Boys	44.89 (4.77)	44.79 (6.14)	43.83 (7.48)	47.39 (6.18)	.248
Girls	47.93 (9.20)	42.40 (5.07)	48.94 (7.85)	46.48 (6.36)	.713

### Qualitative Data

#### Students Theme 1: Enjoyment Levels

Three sub-themes emerged from the analysis of the student’s perceptions of enjoyment of BMT: rules adherence, pedagogy, and perceived self-competence. The respondents felt that student enjoyment levels were enhanced if everyone in the class adhered to the rules of the task or activity. The respondents were also clear that if rules were not adhered to that this increased levels of frustration and prevented a successful experience of BMT:

*“It was really annoying because every time you were with a partner, like some of the partner, well one of my partners was really annoying and he wouldn’t actually do it and then when you got round to doing it he couldn’t actually remember what you were supposed to do.”* (Calvin)

Students commented on the teaching approaches adopted by the teachers, suggesting that the pedagogy used by the staff helped to enhance the student’s experience of BMT and enriched their enjoyment of the subject.

*“.. it’s good cause, when you do the patterns with your partner, Mr Dowens and Mr French don’t say ‘oh, that’s rubbish.’ They’d say, positive things about it and then they’d say something about it that we should work on and that helps so that we know what we can work on next time.”* (Phoebe)

Feedback from the students on their own perceptions of how well they could perform physically in their BMT lessons revealed that if the respondents perceived the task to be too challenging this lowered their levels of enjoyment, whereas, if the perception was that they were good at a specific physical task then this increased their levels of enjoyment.

*“I liked the gymnastics sequences because, if they didn’t tell you what you could do you just could go and do what you were good at, cause if they tell you to do something you might not be good at that so you could show everyone what you were good at and stuff like that and do it well cause you were gonna choose what, like, you’re best at doing.”* (Gemima)

#### Students Theme 2: Perception of What was Learned

Three recurring themes were extracted from analysis of the student’s perceptions of what was learned during their BMT lessons: technical skills, health and fitness and self-confidence. Students fed back that they learned some technical skills in BMT.

*“.. better movers better thinkers taught me to do a forward roll because I couldn’t do a forward roll and then they told me to do this sort of thing where, with my hands up, roll then jump and it made me work better and in every sequence I used a forward roll in.”* (Kjeld)

Health and fitness was commented on by the students, but rather than a direct focus they seemed to perceive it as an indirect outcome from the BMT lessons.

*“Well, I learned that even though exercise can be hard at some points better movers and thinkers gym was always fun and you didn’t really realise that you were exercising all those parts of your body and your muscle but eh, in this time you just thought about having fun and you still improved in your skills that you were doing gym.”* (Monica)

Students mentioned that BMT helped them realise that everyone has different things that they are good at and that it is important to embrace difference. This was clarified with their perception being that self-esteem and social-confidence were improved during BMT sessions.

*“You kinda learned that, just because you can’t do something doesn’t meant that you’re a bad person or that you’re, you’re rubbish at everything but, better movers and thinkers say that, they say that it’s okay not to be good at a handstand but you might be good at something else so say someone could do a handstand and you couldn’t and you were, you feel that you’re rubbish inside and all that, but better movers and thinkers would say well maybe you’re not good at that but you can do something else that they can’t do and it makes you feel better about yourself.”* (Monica)

#### Students Theme 3: Transfer Learning from PE to the Classroom

Students made a link between their bodies and brains both being challenged during BMT stating that *‘[BMT] gets the brain going.’* Student comments were grouped into five different emergent themes which were; BMT gave you more energy for the day in the classroom compared to normal PE;

“Better movers and thinkers is good for class work because when you come to school usually, like see when I look at everybody in my group, including me, are all really tired and we all really need to get warmed up, well, like that’s what better movers and thinkers does, it warms up your body and gets your brain ready for all the work that you’re gonna do and it’s just really helpful for class work.”

BMT exercised your brain, normal PE did not;

“I learned that even though it’s about exercising your body it’s like exercising your brain as well cause it’s like testing it”

After BMT, students felt they worked more efficiently and accurately;

“After I got back from better movers better thinkers I kinda thought like I was so tired, I was like, I was so tired like, I just got changed and I sat down to do my work and I was so tired that I just wanted to kinda get through my work and I knew all the answers and everything and got through my work a lot faster.”

Pupils felt more successful in BMT sessions, which gave more confidence to learn in the classroom;

“I think it was just because after the experience I’d learned a bit and, I got, I’m not sure, I just, I thought I’d learned a bit. It made me a bit more confident in my work.”

Improved concentration and more focus for the next lesson.

“I think it was before better movers and thinkers on a Tuesday we would get, music I think it is, right after gym and Miss Greer would come in and get us and I used to maybe, be like tired or something and I wouldn’t do it properly but now in music on a Tuesday, like, before when, before better movers and thinkers finished, it was really, well, I could concentrate on what I was doing in music and I could, eh we got asked to make or eh compose some eh, eh music on the, eh,…Xylophone and I, I got through it and it was really good.”

#### Class Teachers Theme 1: Classroom Behaviour (PE)

The class teacher reported that despite it being difficult for the children to concentrate for the entire lesson, she noticed that the students were entirely focussed from the beginning to the end of each session, and put this down to the nature of the BMT approach, and felt that the concentration from the pupils improved. She makes specific mention of the girls by stating *‘I think the girls who are maybe a wee bit body conscious at this age…they were totally engaged and involved.’* She outlined that the students would often be working in pairs which helped to develop the pupil’s confidence and that they were willing to try new things. She was particularly interested to note that ‘*the children chose their own partners in the gym and it was, it changed, it wasn’t always the same partner so that was very interesting.’*

#### Class Teacher Theme 2: Links Between BMT and the Class

The class teacher made direct links between BMT and Curriculum for Excellence (Scottish Executive, 2004) recognising the contribution that the sessions made to successful learners, effective contributors, and confident individuals. A further link was made between language development in the classroom and the physical sentence structure that was being developed in BMT, with specific mention of the *‘links and linking words and all that kinda of link, the, the language used and the children tuned in well to that too.’* She then went on to say that the focus and concentration that was developed in BMT raises the pupil’s enthusiasm for learning and that this continues into the classroom and beyond. She specifically recalls seeing the students in the playground doing some of the actions that were part of BMT. Finally, the class teacher made a direct link between the sharing of ideas, appreciation of one another’s work, working in pairs and good demonstrations of good practice as being something that the children do in BMT and in the classroom stating *‘that is something we do in class a lot.’*

#### Class Teacher Theme 3: School Attendance

The class teacher found no connection between school attendances being different on BMT days compared to non-BMT days.

“I don’t think they’ve actually made their attendance better on a PE day as far as I know.”

## Discussion

This study aimed to provide information about the efficacy, acceptability and feasibility of BMT as an approach to delivering PE in primary schools. Two schools received two PE lessons per week over 16 week duration between January and May with both conditions receiving the 32 planned sessions. The control condition received their PE provision from a combination of PE specialist, qualified NGB sports coaches and the class teacher. The intervention condition received all of their BMT sessions from the two experts who had contributed to the design and conception of BMT. The study was completed within the timescale allocated.

The findings from this study revealed statistically significant improved overall score changes in measures of working memory, phonological awareness and segmentation abilities for participants recruited to the BMT intervention condition. Significant group by gender interactions further revealed that boys in the intervention condition made greater gains than boys from the control condition in Phonological Composite Scores, Segments and Spelling subtests.

Traditionally the literature has revealed girls showing advanced language-based skills when compared to boys (Duckworth & Seligman, 2006), yet findings from this exploratory study have shown boys to make more significant gains in scores on certain aspects of language-based tasks when compared to girls as a result of BMT. Therefore, these results are potentially important findings and provide evidence in support of BMT having a positive impact on learning.

The improvements identified in working memory hold specific significance to learning and educational processes as memory is considered the most basic and fundamental concepts required for learning (Brown & Minns, 1999). The statistically significant gains achieved in phonological awareness and segmentation scores are of similar interest to that of the working memory results as these are considered as the foundations of literacy development (Justice & Pullen, 2003). These foundations are known to develop as part of emergent reading and spelling capabilities and the literature has shown that this typically happens in girls ahead of boys (Bruininks, 1978; Ehri et al., 2001; Flynn & Rahbar, 1994; Green & Francis, 1988; Machin & Pekkarinen, 2008). However, the results from this study identified boys making more significant gains and present a different finding from these previous studies. This may be due to the specific focus that BMT has on the development of EF skills; and in particular the enhancement of working memory, inhibitory control and cognitive flexibility. It could be suggested that improvements in EF skills may lead to concomitant improvements in attention and concentration levels as a result of improvements in self-regulatory behaviours. The literature has indicated a direct correlation between increased levels of attention and the development of phonological abilities (Ehri et al., 2001; Green & Francis, 1988). Qualitative findings support this theory as the class teacher made specific reference to the students having to concentrate throughout the duration of the BMT sessions inferring that this is not the case during traditional PE sessions. To substantiate this stance the class teacher remarked how the students remained engaged throughout the BMT session due to the *‘nature of the programme*.’

It has been noted that the influence that improved levels of concentration can have on phonological awareness, and that phonological awareness positively influences spelling (Muter, Hulme, Snowling, & Stevenson, 2004). It may be suggested that the findings from this study indicate that BMT has supported the development of attention and concentration with concomitant influence on academic achievement and in particular a relationship between gains in phonological awareness and improvements in spelling amongst boys. As boys traditionally lag behind girls in this area of development (Duckworth & Seligman, 2006), these findings are interesting and provide evidence in support of BMT having a positive impact on learning.

However, results from the reading tests identified no main effect for the intervention condition suggesting that there has not been a transfer of skills from improved phonological abilities to reading competency as previous research has suggested (Bruininks, 1978; Machin & Pekkarinen, 2008). It may be the duration of the intervention phase was not sufficient enough to allow for transference, or possibly that emergent reading skills develop before the age of this study cohort.

The significant findings from the LASS assessment may have been a result of the levels of Moderate-Vigorous Physical Activity (MVPA) achieved by the intervention condition being greater than that of the control condition, and may perhaps help explain for the gender interaction identified in some of the subtests. As no measurement for MVPA was used during the intervention phase sessions it is not possible to account for the effect of this potential variable. Similarly, no data were collected on the students’ participation in physical activity outside of school, yet many studies have identified this as a key factor when evaluating the impact that physical activity has on academic achievement (Coe et al., 2006; Davis & Cooper, 2011; Etnier, Nowell, Landers, & Sibley, 2006). It could be suggested that limited engagement in MVPA may hinder the development of good coordination and balance control which may limit the direct engagement in physical activity and result in more sedentary behaviours. If there is a link between physical activity and academic achievement, then future studies should take cognisance of this to help account for the differences identified within this pilot study.

The results from the MABC2 identified that balance and postural control scores did not change over the course of this study, as a ceiling effect was observed. These subtests were used as static balance reaches adult levels for open-eye conditions^i^ between 9 and 10 years of age (Wolff et al., 1998) but in retrospect they may not have been sensitive enough for this particular cohort. It has been suggested that static balance maintenance supports the fundamental process of coordinated accurate movements (Nashner, Shumway-Cook, & Marin, 1983) and therefore it could be suggested that any flaws in static balance may limit the student’s access to a worthwhile and positive PE experience as it has the potential to detrimentally affect coordination. Student feedback clearly identified that their enjoyment levels were often linked to their perceived physical competence and therefore poor self-image may result in a disengagement from PE (and physical activity) altogether. Within this study perhaps students with under-developed static balance capabilities were not identified as a symptom of the limitations within the measurement tool used. However, the MABC2 is a popular instrument for the evaluation and identification of children with motor impairment and is used in many clinical and research contexts where studies on validity have shown 80% agreement between the MABC2 and ‘Bruininks-Oseretsky Test of Motor Performance’ (Bruininks, 1978; Chow & Henderson, 2003; Croce, Horvat, & McCarthy, 2001).

Variables that have not been investigated within this study, such as intensity of the physical activity and/or the individual student differences (i.e. motivation) may help explain for the different results. Similarly, no evaluation of the level of teaching expertise or experience was taken into account but may also have impacted upon results.

Findings from the pilot study provide an opportunity for a larger-scaled study to be conducted. Some key adjustments and additions need to be made to the measurement tools used in order to design a more robust study.

### Strengths of the Study

The schools used within the current study shared catchment areas, had a similar school roll and similar PE resources. This helped to reduce the possible influence of other known variables such as socio-economic status (Rhoades, Greenberg, Lanza, & Blair, 2011) and class sizes (Wilson, 2007) from influencing the results.

Both the control and intervention condition received 16 weeks of PE with no omissions. This prevented time spent doing PE being a confounding variable as both conditions received 32 sessions during the 16-week intervention phase.

Testing was completed by the main researcher at both pre- and post-test in the same location within the school for all students. This standardised operational procedures and allowed the students to feel secure within the process. The validity and reliability of both assessment tools used were good for this exploratory study, and both were standardised for a UK population allowing for it to be free from any cultural interpretations as the study took place within Scotland.

Pre-testing was completed in both conditions in December 2011 before the start of the intervention phase in January 2012, and post-testing was completed in both schools within two weeks from the end date of the intervention phase (May 2012). This reduced the influence that time of testing could have had on the data gained in either condition.

The estimate of internal consistency associated with the composite scores for working memory was moderate (α = .61) suggesting good reliability and validity in using a composite score for the two memory subtests.

The teachers and NGB sports coaches delivering the PE experience in either condition were not involved in the collection of the quantitative or qualitative data, or in the analysis of the results. Similarly, the independent researcher who conducted the student focus group and class teacher interview was not involved in the analysis of the quantitative or qualitative data helping to reduce the level of bias as they were blind to the procedures of the intervention and other assessment tools.

### Limitations of the Study

No measurement of the student’s overall level of physical activity was taken at pre- or post-intervention. There is a considerable amount of research that identifies school-aged students who have increased levels of time spent being physically active typically perform better in academic tasks than those who are less physically active (Coe et al., 2006; Davis & Cooper, 2011; Etnier et al., 2006; Trudeau & Shephard, 2010).

There was no measure of the intensity levels of physical activity being achieved by the students during their PE lessons. Studies have shown a different impact on academic performance depending on acute bouts of exercise or chronic bouts of exercise (Ahn & Fedewa, 2011; Biddle & Asare, 2011).

The expertise and experience levels of the teachers as well as their adopted style when delivering PE was not evaluated or compared. Studies (Bailey et al., 2009; Chatoupis, 2009; Demetriou & Höner, 2012) have shown that the approach adopted by the teacher can have a significant effect on the outcomes achieved during the lessons and therefore can have an impact on any benefits associated with this learning opportunity for students. In addition to this, the teachers delivering to the intervention condition contributed to the development of BMT and therefore may have been more motivated to achieve significant results that those who conducted the control condition lessons.

The control condition received their PE provision on Mondays and Thursdays and the intervention condition received their provision on Tuesdays and Thursdays. It may be suggested that the delivery to pupils on different days of the week may have been a confounding variable as their attitudes and motivations to participate in the PE lessons may have been different given that one school received their first session of the week on Mondays (immediately after the weekend) and the other on Tuesdays. This could therefore have had the potential to influence the results realised from the outcome measures.

The measurement tool used for evaluating the physical competence of the students was limited and this restricted the possibility of identifying students with movement difficulties. Therefore, motor incompetence could not be taken into account during the results analysis.

The estimate of internal consistency associated with the composite scores for reading was low (α = .28) and similarly the composite score for phonological subtests was low (α = .44) when conducting and interpreting internal consistency reliability analysis through Cronbach alphas (Sijtsma, 2009).

The reading tests used within LASS 8–11 did not best reflect a pure sight word reading test or cloze reading test as pictures are provided as part of these tests. The illustration represents a non-verbal process and may assist the students in identifying the correct word from 5 possible answers. This does not involve the pupil having to sound out the components of the word itself in order to read it correctly. The word is read out to the child which does result in the child’s need to process the information phonologically, but in the presence of the non-verbal information being provided (i.e. the visual representation of the picture), it reduces the level of reading and phonological processing that has to be done in order to complete this task.

### Conclusion

Quantitative results showed there was an effect between school with the intervention making statistically significant gains in working memory, phonological awareness and segmentation abilities. The quantitative data analysis showed results in spelling that were approaching significance, though the small sample size did not allow for this. There was a group by gender interaction identified and in particular; male students in the intervention condition made significant gains in comparison to the male students in the control condition in measures of phonological awareness, segmentation abilities and spelling. Gains being made, especially for boys, indicate a potential mapping between BMT and academic skills. The gains may be attributed to specific improvements in EF skills, and in particular to inhibitory control, cognitive flexibility and working memory, though further research is required where a direct measure of these core EF skills could be included. This study only included measures of working memory.

Qualitative results indicated that students enjoyed BMT as a different approach to PE and the class teacher felt that it enhanced aspects of classroom learning and in particular the engagement of the girls in PE. The findings support BMT as one approach to PE with concomitant benefits to academic achievement and EF skills.
